# The validation of the Short Warwick-Edinburgh Mental Well-Being Scale (SWEMWBS) with deaf British sign language users in the UK

**DOI:** 10.1186/s12955-018-0976-x

**Published:** 2018-07-24

**Authors:** Katherine D. Rogers, Claire Dodds, Malcolm Campbell, Alys Young

**Affiliations:** Division of Nursing, Midwifery, & Social Work, School of Health Sciences, Faculty of Biology, Medicine and Health, Manchester Academic Health Science Centre, University of Manchester, Manchester, UK

**Keywords:** Mental well-being, Scale validation, SWEMWBS, Deaf population, British sign language

## Abstract

**Background:**

There is no validated measure of positive mental well-being that is suitable for Deaf people who use a signed language such as British Sign Language (BSL). This impedes inclusion of this population in a range of research designed to evaluate effectiveness of interventions. The study aims were: (i) to translate the original English version of SWEMWBS into BSL and to test the SWEMWBS BSL with the Deaf population in the UK who use BSL; (ii) to examine its psychometric properties; and (iii) to establish the validity and reliability of the SWEMWBS BSL.

**Methods:**

The SWEMWBS was translated into BSL following a six stage translation procedure and in consultation with the originators. The draft version was piloted with Deaf BSL users (*n* = 96) who also completed the CORE-OM BSL well-being subscale and the EQ-5D VAS BSL. Reliability was explored using Cronbach’s alpha for internal consistency and ICC for test-retest reliability. Validity was explored by using Kendall’s tau correction for convergent validity and an exploratory factor analysis for construct validity.

**Results:**

The internal consistency for the reliability of the SWEMWBS BSL was found to be good and the test-retest one week apart showed an acceptable reliability. There was good convergent validity of the SWEMWBS BSL with the well-being subscale of the CORE-OM BSL and the EQ-5D VAS BSL.

**Conclusions:**

The SWEMWBS BSL can be used with a Deaf population of BSL users. This is the first validated version of a BSL instrument that focuses solely on positively phrased questions for measuring mental well-being.

## Background

Positive mental well-being supports outcomes at an individual level and is recognised as having social and economic implications in terms of productivity and social cohesion [1]. The New Economics Foundation for example, has outlined five actions to improve mental health and well-being, which are: connect, be active, take notice, keep learning and give [2]. Assessment of mental well-being involves attention to two kinds of features: the hedonic and the eudaimonic [3]. The hedonic features are based on the notion of subjective well-being with affective components (feeling happy) and cognitive ones (e.g. positive appraisal of life satisfaction). The eudaimonic features are based on an approach to well-being that emphasises action, agency and self-actualisation (e.g. sense of control, personal growth, feelings of purpose and belonging). The Warwick-Edinburgh Mental Well-being Scale [4] was designed as a measure encompassing both approaches and focuses entirely on positive mental well-being. The WEMWBS in its English form has been validated with minority hearing populations in the UK who can speak English, such as Chinese and Pakistanis [4]. Additionally, the WEMWBS has been translated into several languages, including Spanish [5], French [6], Norwegian and Swedish [7]. The short version of the Warwick-Edinburgh Mental Well-being Scale [8] (SWEMWBS) is a validated version in UK English. To date there are no versions of either the WEMWBS or the SWEMWBS in BSL or in any other signed language.

Deaf people whose preferred language is a signed language can be considered a minority cultural-linguistic population [9]. Those Deaf people are usually described as Deaf with a capital “D” in the same way as one would mark the cultural identity of other language-using peoples, e.g. Polish not polish [10]. Other people who do not sign but experience hearing loss are conventionally described as ‘deaf’ without capitalisation [11].

It has been estimated that there are between 80,000 and 100,000 Deaf BSL users in England [12]. In comparison with hearing populations, Deaf people experience inequalities in health outcomes and in accessing services [13]. Health-related (including mental health) outcomes for d/Deaf people are generally poorer compared to those of hearing populations [14–16]. Key barriers for health-related equality faced by Deaf people include the lack of information (e.g. about health and positive well-being) and support in their own language [17, 18] and poor awareness by professionals of their linguistic and cultural requirements [19]. In the UK as elsewhere, literacy in a written language remains a key challenge for many Deaf people, as educational attainment lags behind that of hearing peers [20] and unemployment and under-employment remains high [21]. The self-actualisation foundations of positive well-being are consequently quite fragile. That said, one of the central notions of the supportive and positive benefits of a culturally Deaf identity is the opportunity for the sense of belonging, positive contribution, social acceptance and feelings of happiness that may result [22].

At the time of this study there was no BSL measure of positive mental well-being available for the signing Deaf population in the UK. Therefore, the purpose of this study was to translate and validate the SWEMWBS into British Sign Language (BSL) and to test its validity and reliability. The SWEMWBS BSL would then add to the suite of self-reported health-related assessments that are available in BSL (see e.g. [23, 24]. Deaf people are routinely excluded from general population studies because there are no reliable means of assessment and evaluation that would mirror those used for hearing people [25]. Extending the range of assessments in BSL will help include Deaf people in future research studies.

## Methods

### The Short Warwick-Edinburgh Mental Well-Being Scale (SWEMWBS)

The 7 items in the SWEMWBS were originally drawn from the full version of the WEMWBS following Rasch analysis [26] (see Table 2 for the items of the English SWEMWBS). The original version of the WEMWBS contains 14 positively-phrased items covering both hedonic (happiness and life satisfaction) and eudaimonic (psychological functioning and self-realisation) aspects. Each item is scored on a 5-point Likert-type scale ranging from ‘None of the time’ to ‘All of the time’. The raw score calculated as the total across the 7 items, none of which can be absent, is then transformed via a conversion table into a metric score, which should be suitable for parametric analyses [26]. Studies have indicated that the WEMWBS has good validity and reliability for measuring mental well-being in the UK population who use spoken/written English [8]. The items in the SWEMWBS contain more indicators of eudemonic well-being than hedonic well-being. Stewart-Brown et al. (2009) [26] showed that the SWEMWBS was robust in Rasch model analysis, and produced less item bias. The metric score for SWEMWBS ranges from 7 to 35, where the highest score indicates greater mental well-being.

This study was carried out in two phases. The first phase involved translating the original English version of the SWEMWBS into BSL. The second phase involved assessing its reliability and validation.

### Phase one: translating SWEMWBS into BSL

**Fig. 1:**
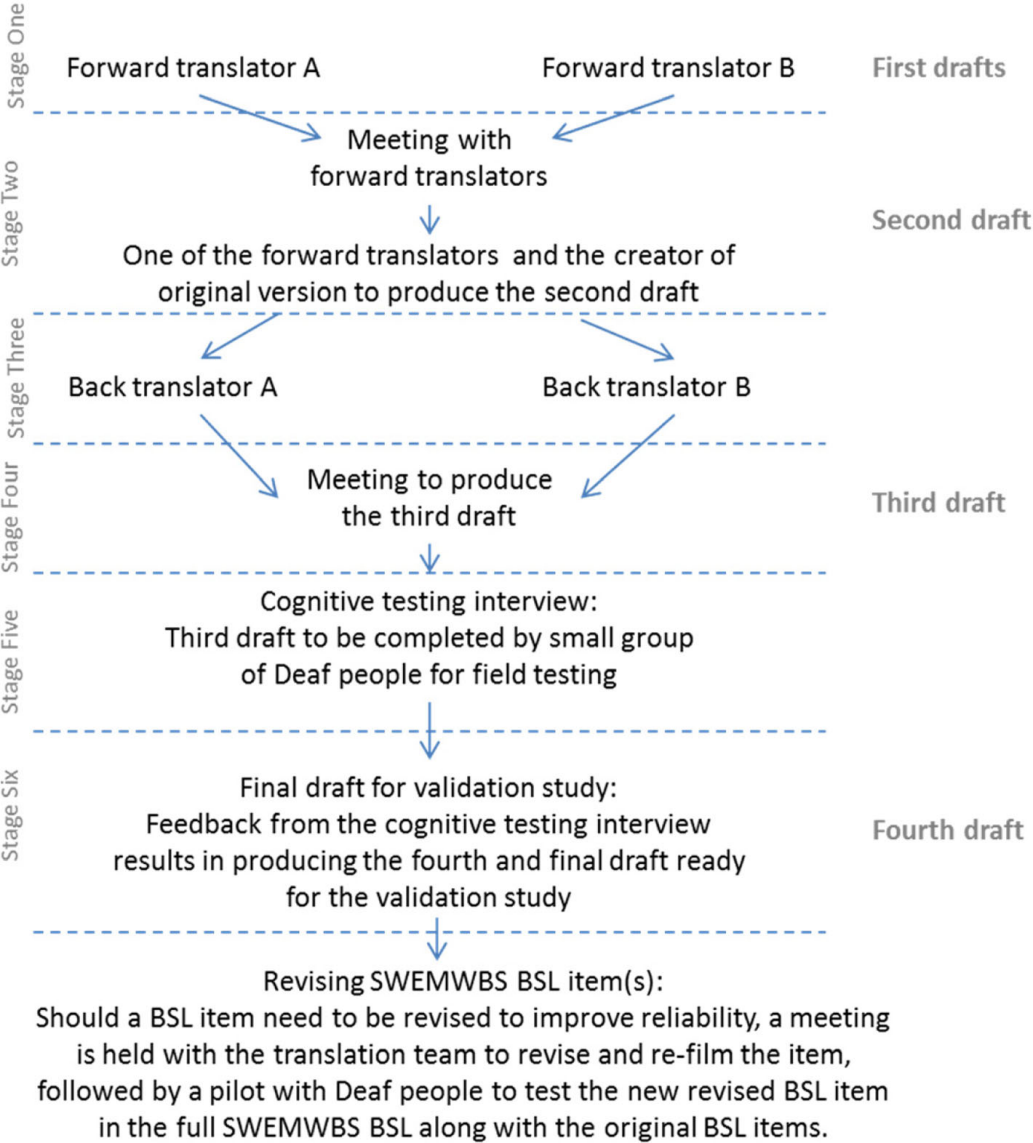
The translation procedure for SWEMWBS into BSL

The translation process follows the requirements of the originators of the SWEMWBS with some minor adaptations because of the use of a visual (not a written) language (i.e. BSL). They gave their permission for the translation and minor adaptations required because of the modality. A general note on the translation process and procedures with respect to written self-report instruments that have been translated into a BSL visual format is available in the journal article [27]. A rigorous translation procedure for translating from an original English version into a sign language version has been carried out previously in other studies [28, 29]; cognitive interviews are included as part of the translation procedure [29, 30]. The aim of this process was to achieve a BSL version conceptually equivalent to the English version. The translation procedure involved the following stages (see Fig. 1):

- Stage One: Two Deaf native BSL users who were bilingually fluent in BSL and written English independently translated the English version into BSL (first drafts). These were the ‘forward translators’. Neither were members of the research team and both were Registered Sign Language Interpreters; in addition, one was a Registered Sign Language Translator^1^ and one was a Trainee Sign Language Translator.
- Stage Two: A meeting was held with the forward translators to resolve any disagreements between their initial first drafts; thus producing the second draft of the BSL version. Frances Taggart, who worked with the (S)WEMWBS team and understood its purpose, attended the meeting via Skype so that the translators had an opportunity to clarify what the questions in their English format aimed to measure.
- Stage Three: Two other Deaf people, who were bilingually fluent in written English and BSL, back translated the second draft of the BSL version into English without looking at the original version. One was a native BSL user who was a Trainee Sign Language Translator at the time of the study, and the other was a Registered Sign Language Interpreter.
- Stage Four: A meeting with all four translators was held to produce the pre-final BSL version (third draft) for field testing.
- Stage Five: The third draft version of the BSL instrument was completed by a small sample (*n* = 27) of Deaf BSL users who had volunteered to participate in cognitive testing interviews. They accessed the 7-item questionnaire in its BSL version via a secure online platform and chose from a set of Likert scale responses from ‘None of the time’ to ‘All of the time’. They did this on only one occasion. Following the completion of the SWEMWBS BSL, participants were interviewed to check their understanding of the questions (question comprehension), how they interpreted the questions and their perception of key words in the questions. For example, they were asked whether the SWEMWBS BSL: (i) was clear and easy to understand; (ii) had clear instructions; and (iii) had any signs that they found difficult to understand. They were also asked (iv) what the questions in the SWEMWBS BSL meant to them; and (v) if they were thinking about a particular aspect of their mental well-being when completing it. Each interview was carried out in BSL, face to face with the Deaf researcher and a volunteer. The interview was also video recorded for the purpose of later analysis. Volunteers were required to do both the test and the interview to be eligible to participate. Upon completion of the interview they were offered a £30 voucher in recompense for their time. A fourth draft was produced after the feedback from the cognitive testing.
- Stage Six (Phase Two): Following completion of the fourth draft, psychometric testing was carried out by a sample of 104 Deaf BSL users to assess the reliability (e.g. internal consistency, test-retest) and validity (e.g. convergent and construct) and responsiveness to change of the BSL version.

### Phase two: testing the validation and reliability of the SWEMWBS BSL

#### Participants

The inclusion criteria required participants to be 18 years or over; self-reported audiologically deaf; BSL users; and able to use an online interface to complete the questionnaire (e.g. not severely visually impaired). The participants who had taken part in stage five (cognitive interview stage) were excluded.

#### Recruitment

Members of the Deaf community were invited to take part in testing out the BSL version of the SWEMWBS via email, Facebook, word of mouth/hands, advertisement in Deaf-related magazines and online message boards.

After completing an online consent form, participants were asked to (i) complete a demographic information sheet which included variables such as age, gender, hearing status of parents, as well as a self-report of current difficulties (if any) with their mental health; (ii) complete the 7 question SWEMWBS in BSL online; and (iii) complete the 4 questions from the CORE-OM BSL (well-being subscale) [24] and the Visual Analogue Scale (EQ-VAS) from the EQ-5D BSL [30] in BSL online (Time 1).

One week later (Time 2), participants were asked to complete the SWEMWBS BSL again. They were prompted to do this by email and SMS and had up to 14 days to respond. The data from the first test was still used in analysis for those participants that did not complete the re-test. Upon completion of the SWEMWBS BSL, validation study participants received £15 in vouchers.

#### Data analysis

Reliability was assessed by internal consistency of the BSL items within the SWEMWBS using Cronbach’s alpha and test-retest using intra-class correlation coefficients (ICCs). Research [8] on the WEMWBS showed an ICC of 0.83 in the adult hearing population which suggested that an ICC of 0.80 in this study might be realistic. A sample size of 51 would allow a 95% confidence interval for an ICC of 0.80 to be estimated with a margin-of-error of ±0.10 [31]. A sample size of 53 would allow a 95% confidence interval for a Cronbach’s alpha of 0.80 to be estimated with a margin-of-error of ±0.10 when there are 3 or more items in a scale [32]. The aim, therefore, was to recruit at least 70 people to allow for attrition of up to 25% at retest. Wilcoxon’s signed-rank test was used to compare item scores between Time 1 and Time 2.

Validity was assessed by examining the convergent validity of the SWEMWBS BSL against the well-being subscale of the CORE-OM BSL and the VAS of the EQ-5D BSL. Kendall’s tau correlation was used as distributions were not normal. The construct validity of the SWEMWBS BSL was assessed using exploratory factor analysis: principal components analysis with Varimax rotation was used to determine whether the SWEMWBS BSL had more than one component. As the planned sample size was small for factor analysis, this analysis was tentative.

The SWEMWBS BSL metric score was compared between sociodemographic groups using independent-samples t-tests and one-way ANOVA. Given the non-normal distributions, scores for the well-being subscale of the CORE-OM BSL and the VAS of the EQ-5D BSL were compared between groups using the Mann-Whitney U test and the Kruskal-Wallis test.

#### Ethical approval

Ethical approval was required for the cognitive interviews and for testing the reliability and validation of SWEMWBS BSL and was granted through the University of Manchester Research Ethics Committee (ref: 150616).

## Results

### Cognitive testing interview

The third draft of the pre-validation SWEMWBS BSL was tested with 27 Deaf participants (14 females and 13 males; aged between 26 to 76 years old). Six participants self-reported experiencing current difficulties with their mental health via an online survey. Participants were interviewed in BSL to check their understanding of the questions (question comprehension) and how they interpreted the questions and the key signs in the questions. Most of the participants were able to explain the key concepts conveyed in the BSL items (e.g. ‘feeling optimistic’; and ‘feeling useful’). There was a discussion about how best to improve the BSL questions, where some participants commented on the need to make the question clearer by expanding on the questions in a negative way (e.g. ‘thinking clearly’ as in ‘without having muddled up thoughts in your head’). The challenge for them (as well as for the translation team) was to ensure that all BSL items were expressed as a positive phrase, as requested by the creators of the SWEMWBS. How Deaf participants understood and conceptualised the key BSL items, along with the examples given when answering questions, was influenced by their own experiences of being a Deaf person e.g. relating to communicating, accessibility and mixing with others. For example, when considering the question about being able to deal with problems well, a few participants speculated whether the very solutions to a particular problem were communicatively accessible to them in the first place, thereby influencing their answer to the question. When discussing the question relating to feeling close to other people, participants commented that it depended on who the ‘other’ people are, i.e. they would answer differently depending on whether it referred to Deaf or hearing people and whether they could communicate with them or not. Feedback from the participants was taken into consideration when the revisions for the fourth draft were made. Examples of revisions included: (i) changing the structure for each question so that each question started with ‘In the last two weeks have you been feeling … etc.’; (ii) more positive facial expression for each of the statements; and (iii) signs used for some of the key words, e.g. ‘dealing with’ and ‘thinking clearly’, were amended to make them clearer.

### Psychometric properties of the fourth draft of the SWEMWBS BSL

In total, 104 participants took part in Stage Six - assessing the reliability and validity of the BSL version of the SWEMWBS. Four participants were not included in the data analysis, as one had already taken part in the previous stage of this study (respondent testing stage), one provided incomplete data, one participant was hard of hearing and fell outside of the inclusion criteria, and the consent for one participant was missing. Additionally, for the data analysis for the validation of the SWEMWBS BSL, participants would need to have completed all 7 items. Four further participants were missing one of the 7 items for the SWEMWBS BSL (missing individual BSL items: No.1, No. 2, No. 6, and No.7), which left a total of 96 participants to be included in the data analysis.

#### Demographic information

Most of the participants at Time 1 were female (*n* = 66, 68.8%), 29 were male (30.2%) and 1 was trans (1.0%). The age of the participants ranged from 19 to 79, with the mean age being 42 years, and the median being 41 years. Table 1 gives demographic information on the participants, 84 of whom also participated at Time 2. There were no differences in the characteristics of those participating at Time 1 and those participating at Time 2.

**Table 1:** Demographic details of participants

	Time 1 (test) *N* = 96	Time 2 (retest) *N* = 84
Gender		
Female	66 (68.8%)	56 (66.7%)
Male	29 (30.2%)	27 (32.1%)
Trans	1 (1.0%)	1 (1.2%)
Age		
18–24	8 (8.3%)	6 (7.1%)
25–34	19 (19.8%)	17 (20.2%)
35–44	29 (30.2%)	25 (29.8%)
45–54	24 (25.0%)	21 (25.0%)
55–64	8 (8.3%)	7 (8.3%)
65+	6 (6.3%)	6 (7.1%)
Missing data	2 (2.1%)	2 (2.4%)
Ethnicity		
Asian or Asian British: Indian	1 (1.0%)	1 (1.2%)
Asian or Asian British: Pakistani	1 (1.0%)	0 (0%)
Asian or Asian British: Other Asian background	1 (1.0%)	1 (1.2%)
Black or Black British: African	1 (1.0%)	1 (1.2%)
Black or Black British: Other Black background	2 (2.1%)	2 (2.4%)
Mixed: Any other mixed background	2 (2.1%)	2 (2.4%)
Other ethnic group	3 (3.1%)	3 (3.6%)
White: British	74 (77.1%)	64 (76.2%)
White: Irish	3 (3.1%)	2 (2.4%)
White: Any other white background	3 (3.1%)	3 (3.6%)
Missing data	5 (5.2%)	5 (6.0%)
Parents d/Deaf?		
Yes	29 (30.2%)	25 (29.8%)
No	67 (69.8%)	59 (70.2%)
Age first used BSL		
From birth – 3 years old	41 (42.7%)	37 (44.0%)
4–7 years old	12 (12.5%)	11 (13.1%)
8–11 years old	7 (7.3%)	6 (7.1%)
12–16 years old	18 (18.8%)	14 (16.7%)
17–24 years old	11 (11.5%)	10 (11.9%)
25+ years old	7 (7.3%)	6 (7.1%)
Currently in employment		
Yes	59 (61.5%)	51 (60.7%)
No	36 (37.5%)	32 (38.1%)
Missing data	1 (1.0%)	1 (1.2%)
Mental health difficulties		
Yes	19 (19.8%)	15 (17.9%)
No	63 (65.6%)	56 (66.7%)
I don’t know	14 (14.6%)	13 (15.5%)

At least half of the participants (*n* = 51) had family members who were d/Deaf (53.1%); 29 had d/Deaf parent(s), 25 of whom reported that both parents were d/Deaf. Other family members who were d/Deaf included sibling(s) (*n* = 28), as well as grandparents, aunts or uncles, cousins, children and nieces or nephews. Of those with family members who were d/Deaf, 41 reported that their family members were culturally Deaf.

Twelve considered themselves as having a disability, other than being deaf. Self–reported disabilities included autism, borderline personality disorder, diabetes, functional neurological disorder, monopolar depression, arthritis and visual impairments. With regards to the self-report of current mental health difficulties, the majority (*n* = 63, 65.6%) reported that they did not currently have mental health difficulties whereas 19 (19.8%) reported that they did and 14 (14.6%) reported that they did not know whether they were currently experiencing mental health difficulties or not. Among those who stated what mental health difficulties they had, most difficulties were related to depression and/or anxiety. Nearly half (*n* = 9, 47.4%) of those who reported they had current mental health difficulties also reported that they had long-standing mental health difficulties.

#### Test for normality: the SWEMWBS BSL, CORE-OM well-being subscale BSL, and EQ-5D VAS BSL

The Kolmogorov-Smirnov test for the normality of the SWEMWBS BSL metric score at Time 1 indicated a deviation from normality (*D*(96) = 0.12, *p* = 0.001), although a histogram appeared to be reasonably normal. Deviations from normality were also found for the well-being subscale of CORE-OM BSL (*D*(95) = 0.11, *p* = 0.006) and for EQ-5D VAS BSL (*D*(94) = 0.14, *p* <  0.001). Their histograms showed clear positive and negative skewness respectively.

#### Reliability

##### Internal consistency

Cronbach’s alpha was α = 0.83 for the raw score of the SWEMWBS BSL at Time 1 and α = 0.85 for the raw score of the retest SWEMWBS BSL at Time 2, which indicated a good reliability. For the well-being subscale of CORE-OM BSL, Cronbach’s alpha was 0.80. SWEMWBS BSL item 1 had correlations with three other items (2, 5 and 6) that were less than 0.3. If SWEMWBS BSL item 1 were to be removed, however, then the α value would improve only slightly, by 0.001.

##### Test-retest reliability

For participants taking part at both time points, the SWEMWBS BSL metric score at Time 1 (mean = 22.82, SD = 4.67) and Time 2 (mean = 23.40, SD = 4.41) had an ICC of 0.72 with 95% CI (0.60, 0.81). The test-retest reliability of individual SWEMWBS BSL items is presented in Table 2. The lowest ICC value for an individual item was that for item 1 (ICC = 0.30, with 95% CI (0.10–0.48), *p* = 0.002).

**Table 2:** Test-retest reliability for the SWEMWBS BSL (*n* = 84)

SWEMWBS BSL	ICC	ICC 95% CI	*P*
Metric score	0.72	0.60–0.81	< 0.001
Item 1: I’ve been feeling optimistic about the future	0.30	0.10–0.48	< 0.002
Item 2: I’ve been feeling useful	0.53	0.36–0.67	< 0.001
Item 3: I’ve been feeling relaxed	0.59	0.44–0.72	< 0.001
Item 4: I’ve been dealing with problems well	0.40	0.21–0.57	< 0.001
Item 5: I’ve been thinking clearly	0.48	0.30–0.63	< 0.001
Item 6: I’ve been feeling close to other people	0.57	0.41–0.70	< 0.001
Item 7: I’ve been able to make up my own mind about things	0.50	0.32–0.64	< 0.001

A cross-tabulation of SWEMWBS BSL item 1 at Time 1 vs Time 2 showed that 44 out of 87 participants gave the same response at Times 1 and 2. A Wilcoxon signed-rank test showed that the SWEWMBS BSL item 1 did not change significantly between Time 1 and Time 2 (Z = − 1.058, *p* = 0.290). It is interesting to note that two SWEMWBS BSL items (item 3 and item 6) showed significant changes in response between Time 1 and Time 2 (Z = − 2.205, *p* = 0.027 for item 3, and Z = − 1.969, *p* = 0.049 for item 6) although they did not show clear evidence of non-reliability.

##### Length of time between time 1 and time 2 in days

**Fig. 2:**
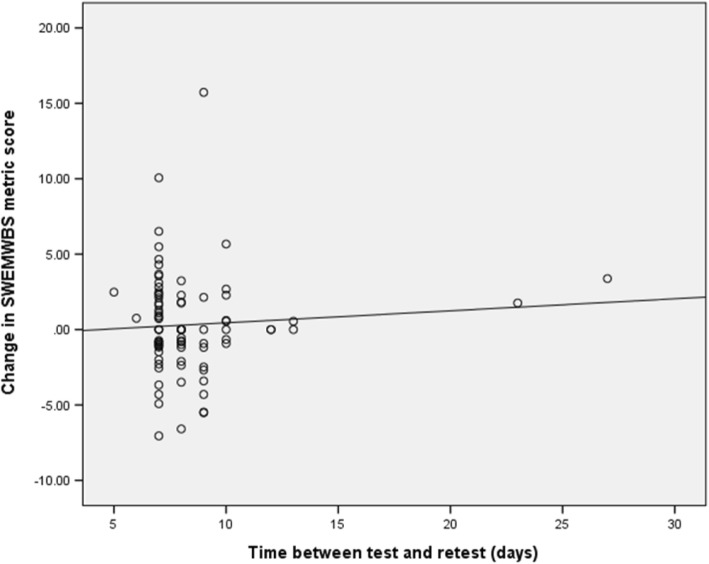
Scatter plot of the change in the SWEMWBS metric score against time (days) between Time 1 and Time 2

The duration between Time 1 and Time 2 for the majority of participants was 7 ± 2 days (*n* = 71, 82.6%) and nearly all participants completed the retest in under two weeks (82 out of 84, 97.7%). A relationship was explored between the number of the days’ duration between Time 1 and Time 2 and the change in the SWEMWBS BSL metric score (see Fig. 2 for a scatter plot between the two variables). This showed a very slight but non-significant increase in mean SWEMWBS BSL metric score shown by the regression line as time increased between Time 1 and Time 2. This was accounted for by the two outliers; there was an even scatter of points about zero for participants whose retest took place within two weeks.

#### Validity

##### Convergent validity

Using Kendall’s tau correlation, the association between the SWEMWBS BSL metric score at Time 1 and the CORE-OM BSL well-being subscale showed a negative correlation of *τ* = − 0.605 (*p* <  0.001). The correlation between the SWEMWBS BSL and EQ-5D VAS BSL was positive (*τ* = 0.414, *p* <  0.001). High SWEMWBS BSL scores, low CORE-OM BSL scores and high EQ-5D VAS BSL scores correspond to better mental well-being and the significant correlations in the expected directions demonstrated convergent validity for the SWEMWBS BSL metric score.

##### Construct validity

Principal components analysis was carried out to assess the viability of a one component solution for the seven items of the SWEMWBS BSL at Time 1. The first principal component had an eigenvalue of 3.48 and accounted for 49.7% of the total standardised variance. The second principal component increased the amount of standardised variance explained to 63.3%; it had an eigenvalue of 0.95, just under Kaiser’s cut-off of 1.00, and the scree plot also suggested a one-component solution. The loadings for the SWEMWBS BSL items on the first principal component scores (Pearson correlations between items and the component) ranged from 0.55 to 0.79 which is considered to be high. The KMO measure of sampling adequacy was 0.855 (also high).

A two factor solution was explored using principal axis factoring with Varimax rotation. From the rotated factor loadings there was not a clear separation of items into the two factors, with items 2, 3, 6 and 7 more strongly associated with the first factor, items 1 and 4 more strongly associated with the second factor and item 5 split between the two. The loading of the potentially problematic item 1 on the second factor was only 0.53 compared with 0.55 on the first principal component. There was no evidence that a two factor solution was better than the solution based on the first principal component and it was concluded tentatively (given the relatively small sample size for factor analysis) that the SWEMWBS BSL was unidimensional.

##### Analysis of well-being measures at time 1 (test)

The mean for the metric score of SWEMWBS BSL for all participants at Time 1 was 22.82 (*n* = 96, SD = 4.67, 95% CI = 21.88–23.77). The mean score for Deaf participants in this study was slightly lower than was reported in the study by Fat et al. (2017) [33] for the general hearing population for men (23.7) and for women (23.6) in England, although the Fat et al. (2017) [33] overall population mean of 23.64 was near the upper end of the 95% CI for this study mean (Upper 95% CI = 23.77). The CORE-OM BSL well-being subscale had a mean score of 1.35 (SD = 0.93, *n* = 95), and the EQ-5D VAS BSL had a mean score of 68.0 (SD = 21.18, *n* = 94).

##### Report of current mental health difficulties

Table 3 shows a breakdown of scores for the three measures of mental well-being according to whether the participants reported current mental health difficulties or not. For all three measures, mean scores reflected the degree to which participants were currently experiencing mental health difficulties: those who were experiencing mental health difficulties had the worst mean score and those who were not had the best mean score for the respective scale.

**Table 3:** SWEMWBS BSL metric score, CORE-OM BSL well-being subscale and EQ-5D VAS BSL by whether participants were currently experiencing mental health difficulties

	N	Mean (SD)
SWEMWBS BSL metric score		
Currently experiencing mental health difficulties	19	19.37 (2.61)
I don’t know	14	21.51 (4.44)
No mental health difficulties	63	24.16 (4.63)
CORE-OM BSL Well-being subscale		
Currently experiencing mental health difficulties	18	2.40 (0.72)
I don’t know	14	1.45 (0.75)
No mental health difficulties	63	1.03 (0.78)
EQ-5D VAS BSL		
Currently experiencing mental health difficulties	18	51.22 (17.71)
I don’t know	14	66.50 (20.59)
No mental health difficulties	62	73.24 (19.86)

One-way ANOVA showed that there was a significant difference in the self-reporting of current mental health difficulties on the SWEMWBS BSL metric score (*F*(2, 93) = 9.89, *p* <  0.001). A Tukey post hoc test showed that those who were currently experiencing mental health difficulties had a significantly lower (worse) mean score compared to those who were not currently experiencing mental health difficulties (mean difference = − 4.79, *p* <  0.001).

For the well-being subscale score of the CORE-OM BSL, a Kruskal-Wallis test showed that there were differences in the distributions of scores between the three groups (K-W χ^2^ = 28.36, df = 2, p <  0.001). Using Mann-Whitney U tests with a Bonferroni correction, there were significant differences in distribution between those experiencing and those not experiencing mental health difficulties (corrected *p* < 0.001) and those experiencing mental health difficulties and those who were not sure (corrected *p* = 0.032).

The EQ-5D VAS BSL score also showed a significant difference between groups (K-W χ^2^ = 17.50, df = 2, *p* < 0.001). Again using a Mann-Whitney U test with a Bonferroni correction, there was a significant difference in distribution between those experiencing and those not experiencing mental health difficulties (corrected *p* < 0.001).

##### Gender

There were 66 females, 29 males and 1 trans person who took part in the study. As can be seen in Table 4, males and females had identical sample means for the SWEMWBS BSL metric score.

**Table 4:** SWEMWBS BSL metric score by gender, whether in employment and being culturally Deaf

	N	Mean (SD)
Gender		
Female	66	22.81 (4.70)
Male	29	22.81 (4.78)
In employment		
Yes	59	23.40 (4.29)
No	36	21.54 (4.72)
Culturally Deaf		
Somewhat / Quite so / Very much so	85	23.01 (4.59)
Not at all / A little bit	6	20.46 (3.10)

##### Employment

There were 59 participants who reported that they were currently in employment and 36 who reported that they were not in employment. From Table 4, those currently in employment had a slightly higher mean SWEMWBS BSL metric score. Mean scores just failed to be significantly different by whether or not the participant was currently in employment (*t*(93) = − 1.97, *p* = 0.052, *r =* 0.42). However, when excluding the 4 who were 66 years old or over,^2^ 59 participants reported that they were currently in employment and 32 were not. The mean score for those not in employment was 21.10 (SD = 4.68), was significantly different to the mean for those in employment (mean = 23.40, SD = 4.29) (*t*(89) = − 2.34, *p* = 0.020, *r =* 0.52).

##### Culturally deaf

One of the inclusion criteria was whether the individual was a BSL user; however, whether they considered themselves to be culturally Deaf or not is a personal judgement. Of 91 participants who reported whether they were culturally Deaf or not, 85 reported that they were ‘somewhat’, ‘quite so’ or ‘very much so’ culturally Deaf and 6 reported being ‘Not at all’ or ‘A little bit’ culturally Deaf. As shown in Table 4, the first group had a slightly higher mean SWEMWBS BSL metric score although the difference was not significant (t(89) = − 1.33, *p* = 0.186, *r* = 0.56).

### Phase three: Pilot with deaf people to test the new revised BSL item of the SWEMWBS BSL

The analysis of the reliability of the SWEMWBS BSL items confirmed that all BSL items were reliable. However, there was one BSL item that could possibly have been made clearer in order to slightly improve its reliability. The revision of this item (SWEMWBS BSL No. 1), which involved a slight change to the structure of the BSL signing, was made following meetings with the translation team, and it was agreed that the new revised SWEMWBS BSL item no. 1 was clearer than the original BSL item no. 1. The new revised BSL item no. 1 was then tested with 66 Deaf people who took part in Phase Two (testing the reliability stage) to see whether the revised BSL item had improved the reliability of the SWEMWBS BSL. An amendment to the original ethical approval was granted to enable this extension into Phase Three to occur.

In total, 66 Deaf people completed Phase Three which included a revised version of the SWEMWBS BSL. This started with a new version of item 1, followed by previous version of items 2–7 to complete the scale, followed by the original version of item 1 to compare against the new version. The Cronbach’s alpha for the raw score of the SWEMWBS BSL with the new revised BSL item no. 1 was α = 0.84, which indicates a good reliability; this lay between the reliability for the SWEMWBS BSL with the original version of item no. 1 at Time 1, which was α = 0.83 and that at Time 2, which was α = 0.85. There were no BSL items that would improve the Cronbach’s alpha value for the internal consistency of the BSL items if they had been removed. The results from a dependent-samples t-test showed that Deaf people’s answer to the revised item no. 1 did not significantly differ to their answer to the original item no. 1 (*t*(65) = − 1.654, *p* = 0.103, *r* = 0.20).

## Discussion

The results show the robust psychometric properties of the SWEMWBS BSL. This indicates that it can be used to measure positive mental well-being in Deaf adults who use BSL in the UK. The internal consistency for the reliability of the SWEMWBS BSL items as measured by Cronbach’s alpha was found to be good (an alpha value of 0.8 or greater), as defined by George and Mallery (2003) [34]. Test-retest approximately one week apart showed an acceptable reliability, indicating that participants’ initial responses to the SWEMWBS BSL questions were consistent with their responses about one week later. The SWEMWBS BSL showed good convergent validity with the well-being subscale of the CORE-OM BSL and the EQ-5D VAS BSL. The negative association between the scores for the SWEMWBS BSL and the CORE-OM BSL well-being subscale was found to be significant, where a low score for the CORE-OM BSL well-being subscale and a high score for the SWEMWBS BSL both indicate a better mental well-being. The SWEMWBS BSL and the EQ-5D VAS BSL had a significant positive association, where high scores on both indicate a better mental well-being. For the construct validity, in line with the other different language versions of the SWEMWBS, the SWEMWBS BSL showed a one component solution (e.g. Tennant et al., 2007 [8], for the original English version).

Further analysis of the SWEMWBS BSL scores from Deaf participants in this study has raised some interesting findings. There are two variables that appear to influence mental well-being scores within the Deaf population: (i) a report of current mental health difficulties (including a report of depression and/or anxiety); and (ii) employment. Employment status and mental health have also been reported as factors which affect mental well-being in hearing populations [35]. Those who reported having current mental health difficulties had significantly poorer mental well-being compared to those who were not currently experiencing mental health difficulties, based on the SWEMWBS BSL score, the well-being subscale of CORE-OM BSL and the EQ-5D VAS BSL score. For those who were aged under 66 years old, there were differences in the well-being score between those who were in employment and those who were not. Based on the mean SWEMWBS BSL score, those who were not in employment had significantly lower mental well-being compared to those who were in employment. Given that Deaf people experience greater unemployment than their hearing peers [21] this is an important finding indicating how mental well-being might be improved for this population. However, no data were taken on whether those who were unemployed were or were not engaged in other non-paid productive activity such as volunteering or unpaid employment roles. Therefore it is not clear whether it is paid employment (including the economic benefits) and/or regular productive activity per se that is explanatory.

As reported in Fat et al. (2017) [33] for the SWEMWBS score in the hearing population, this study found no difference in the mean SWEMWBS BSL score between females and males. The mean SWEMWBS score for males and females combined in the very large study in the hearing population by Fat et al. (2017) [33] was just below the upper end of the 95% confidence interval for the mean SWEMWBS BSL score in this study. Additionally, this study found no difference in the mean SWEMWBS BSL score according to the extent to which participants considered themselves to be culturally Deaf. This might indicate that a strong self-identification as culturally Deaf on its own is not a key factor for positive mental well-being; other associated secondary factors might be such as a sense of belonging to the Deaf community [36] and a self-acceptance of being d/Deaf [37].

The final version SWEMWBS BSL included a revised BSL item no. 1 that was used in Phase Three. The revised version of item no. 1 was felt to be clearer by the translation team and, when combined with the other six items, showed the same internal consistency as the previous version of the SWEMWBS BSL including the original version of item no. 1.

The currently available assessments of the mental health status amongst Deaf people in the UK which are validated and reliable, such as the PHQ-9 BSL, GAD-7 BSL [23] and CORE-OM BSL [24] provide clinical indications of a disorder that would benefit from treatment and intervention. This has been important in the development of better primary mental health services given that the prevalence of mental health difficulties in the Deaf population in the UK is higher than that in hearing populations (e.g. higher rate of anxiety and depression [16]. However, such assessments are unable to gauge positive mental well-being which is important as this can be a good indicator of how well people can function in everyday life and the realisation of their own capacities [38]. It has been recognised that mental health is vital for growth, development, learning and resilience [39]. The association between positive well-being and learning has been reported in some studies [40, 41]. This is of importance in a population who experience less than optimal outcomes in a range of domains including education and employment.

A validated BSL version of the SWEMWBS to measure positive mental well-being amongst Deaf people in the UK will be a useful tool for those who want to evaluate the impact of projects and interventions with the Deaf population. This includes projects which, for example, focus on learning and social engagement where a measure of mental well-being may be an important outcome indicator of effectiveness. A non-clinical mental measure is also of value in measuring responsiveness to change for projects focussing directly on increasing mental well-being.

## Conclusion

Although there is a great need for better approaches to the assessment and treatment of mental ill health amongst signing Deaf people, there is also a parallel need to discover what enables positive well-being and better quality of life for this largely socially excluded, economically disadvantaged population. A valid means of measurement of well-being is a key component in this ambition to promote positive mental health with, by and for Deaf people. This work has produced the first validated version of a positive mental well-being standard instrument in any signed language in the world. It is made freely available to potential users in line with the originators’ requirements. Enquiries may be addressed to the first author.

## Footnotes

1 Like Registered Sign Language Interpreters, a Registered Sign Language Translator is highly skilled in British Sign Language and another language (e.g. English), has completed an approved training course, and is registered with an approved registration body, namely the National Register of Communication Professionals working with Deaf and Deafblind People (NRCPD).

2 They were of retirement age.

## References

[1] Department of Health. Wellbeing: Why it matters to health policy? Health is the top thing people say matters to their wellbeing. Department of Health. 2014; https://www.gov.uk/government/uploads/system/uploads/attachment_data/file/277566/Narrative__January_2014_.pdf. Accessed 6 Nov 2017

[2] Aked J, Thompson S (2011). Five ways to wellbeing: new applications, new ways of thinking. London. England: NEF.

[3] Ryan RM, Deci EL (2001). On happiness and human potential: a review of research on hedonic and eudaimonic well-being. Annu Rev Psychol.

[4] Taggart F, Friede T, Weich S, Clarke A, Johnson M, Stewart-Brown S (2013). Cross cultural evaluation of the Warwick-Edinburgh mental well-being scale (WEMWBS) – a mixed methods study. Health Qual Life Outcomes.

[5] Lopez MA, Gabilondo A, Codony M, Garcia-Forero C, Vilagut G, Castellvi P (2013). Adaptation into Spanish of the Warwick-Edinburgh mental well-being scale (WEMWBS) and preliminary validation in a student sample. Qual Life Res.

[6] Trousselard M, Steiler D, Dutheil F, Claverie D, Canini F, Fenouillet F (2016). Validation of the Warwick-Edinburgh mental well-being scale (WEMWBS) in French psychiatric and general populations. Psychiatry Res.

[7] Haver A, Akerjordet K, Caputi P, Furunes T, Magee C (2015). Measuring mental well-being: a validation of the Short Warwick-Edinburgh Mental Well-Being Scale in Norwegian and Swedish. Scandinavian Journal of Public Health.

[8] Tennant R, Hiller L, Fishwick R, Platt S, Joseph S, Weich S (2007). The Warwick-Edinburgh mental well-being scale (WEMWBS): development and UK validation. Health Qual Life Outcomes.

[9] Ladd P (2003). Understanding Deaf Culture. Search of Deafhood.

[10] Young A, Temple B (2014). Approaches to social research: the case of deaf studies.

[11] Woodward J (1972). Implications for sociolinguistics research among the deaf. Sign Language Studies.

[12] BDA. What is BSL? British deaf association. 2016. https://bda.org.uk/help-resources/#BSL. Accessed 19 Sept 2017.

[13] Rogers KD, Ferguson-Coleman E, Young A (2018). Challenges of Realising patient-centered outcomes for deaf patients. The Patient - Patient-Centered Outcomes Research.

[14] Barnett S, Klien JD, Pollard R, Samar V, Schlehofer D, Starr M (2011). Community participatory research with deaf sign language users to identify health inequities. Am J Public Health.

[15] SignHealth. A report into the health of deaf people in the UK. London: SignHealth. 2014. https://www.signhealth.org.uk/sick-of-it-report-professionals/. Accessed 21 Aug 2017.

[16] Joint Commissioning Panel for Mental Health, SignHealth. Guidance for Commissioners of Primary Care Mental Health Services for Deaf People (2017). Practical mental health commissioning.

[17] Alexander A, Ladd P, Powell S (2012). Deafness might damage your health. Lancet.

[18] Fellinger J, Holzinger D, Pollard, R. Mental health of deaf people. The Lancet 2012;379(9820):1037–1044. 10.1016/S0140-6736(11)61143-4. 2012. 10.1016/S0140-6736(11)61143-4 22423884

[19] NHS England. Principles for high quality interpreting and translation services. Version 1.19. 2015. https://www.england.nhs.uk/commissioning/wp-content/uploads/sites/12/2015/03/it_principles.pdf. Accessed 21 Aug 2017.

[20] NDCS (2016). New GCSE figures show extensive attainment gap for deaf children.

[21] RNID (2003). Employment for all: assisting people with health problems and disabilities into work. Submission by RNID to the Work and Pensions Committee.

[22] Rogers KD (2013). Deaf people and mental well-being: Exploring and measuring mental well-being in British sign language. PhD thesis.

[23] Rogers KD, Young A, Lovell K, Campbell M, Scott PR, Kendal S (2013). The British sign language versions of the patient health questionnaire, the generalized anxiety disorder 7-item scale, and the work and social adjustment scale. J Deaf Stud Deaf Educ.

[24] Rogers KD, Evans C, Campbell M, Young A, Lovell K (2014). The reliability of British sign language and English versions of the clinical outcomes in routine evaluation – outcome measure with d/deaf populations in the UK: a pilot study. Health & Social Care in the Community.

[25] Young A, Rogers K, Davies L, Pilling M, Lovell K., Pilling S, et al. Evaluating the effectiveness and cost-effectiveness of British sign language improving access to psychological therapies: an exploratory study. Health Services and Delivery Research 2017;5:24. https://www.journalslibrary.nihr.ac.uk/programmes/hsdr/1213679/#/. Accessed 19 Sept 2017. 28880505

[26] Stewart-Brown S, Tennant A, Tennant R, Platt S, Parkinson J, Weich S (2009). Internal construct validity of the Warwick-Edinburgh mental well-being scale (SWEMWBS): a Rasch analysis using data from the Scottish health education population survey. Health Qual Life Outcomes.

[27] Rogers KD, Young A, Lovell K, Evans C (2013). The challenges of translating the clinical outcomes in routine evaluation – outcome measure (CORE-OM) into British sign language. J Deaf Stud Deaf Educ.

[28] Cornes AJ, Brown PM (2012). Mental health of Australian deaf adolescents: an investigation using an Auslan version of the strengths and difficulties questionnaire. Deafness & Education International.

[29] Graybill P, Aggas J, Dean RK, Demers S, Finigan EG, Pollard RQ (2010). A CommunityParticipatory approach to adapting survey items for deaf individuals and American sign language. Field Methods.

[30] Rogers K, Pilling M, Davies LM, Belk R, Nassimi-Green C, Young A (2016). Translation, reliability and validity of the British sign language (BSL) version of the EQ-5D-5L. Qual Life Res.

[31] Bonett DG (2002). Sample size requirements for estimating intraclass correlations with desired precision. Stat Med.

[32] Bonett DG (2002). Sample size requirements for testing and estimating coefficient alpha. J Educ Behav Stat.

[33] Fat LN, Scholes S, Boniface S, Mindell J, Stewart-Brown S (2017). Evaluating and establishing national norms for mental wellbeing using the Short Warwick-Edinburgh Mental Well-Being Scale (SWEMWBS): findings from the health survey for England. Qual Life Res.

[34] George D, Mallery P (2003). SPSS for windows step by steps: a simple guide and reference.

[35] Chanfreau J, Lloyd C, Byron C, Roberts C, Craig R, De Feo D, McManus S. Predicting wellbeing. London: NatCen. Soc Res. 2013; http://www.natcen.ac.uk/media/205352/predictors-of-wellbeing.pdf. Accessed 14 May 2018

[36] Sheppard K, Badger T (2010). The lived experience of depression among culturally deaf adults. J Psychiatr Ment Health Nurs.

[37] Griggs M (1998). Deafness and Mental Health: Perceptions of health within the deaf community. PhD thesis.

[38] Ryff CD (2014). Psychological well-being revisited: advances in the science and practice of Eudaimonia. Psychother Psychosom.

[39] Faculty of Public Health and Mental Health Foundation. Better Mental Health for All (2016). A public health approach to mental health improvement. London: faculty of public health and mental Health Foundation.

[40] Feinstein L, Hammond C (2004). The contribution of adult learning to health and social capital. Oxf Rev Educ.

[41] Feinstein L, Vorhaus J, Sabates R (2008). Learning through life challenge report.

